# The shape of things to come: Topological data analysis and biology, from molecules to organisms

**DOI:** 10.1002/dvdy.175

**Published:** 2020-04-13

**Authors:** Erik J. Amézquita, Michelle Y. Quigley, Tim Ophelders, Elizabeth Munch, Daniel H. Chitwood

**Affiliations:** ^1^ Department of Computational Mathematics, Science & Engineering Michigan State University East Lansing Michigan USA; ^2^ Department of Horticulture Michigan State University East Lansing Michigan USA; ^3^ Department of Mathematics Michigan State University East Lansing Michigan USA

**Keywords:** biology, data science, mathematical biology, persistent homology, shape, topological data analysis

## Abstract

Shape is data and data is shape. Biologists are accustomed to thinking about how the shape of biomolecules, cells, tissues, and organisms arise from the effects of genetics, development, and the environment. Less often do we consider that data itself has shape and structure, or that it is possible to measure the shape of data and analyze it. Here, we review applications of topological data analysis (TDA) to biology in a way accessible to biologists and applied mathematicians alike. TDA uses principles from algebraic topology to comprehensively measure shape in data sets. Using a function that relates the similarity of data points to each other, we can monitor the evolution of topological features—connected components, loops, and voids. This evolution, a topological signature, concisely summarizes large, complex data sets. We first provide a TDA primer for biologists before exploring the use of TDA across biological sub‐disciplines, spanning structural biology, molecular biology, evolution, and development. We end by comparing and contrasting different TDA approaches and the potential for their use in biology. The vision of TDA, that data are shape and shape is data, will be relevant as biology transitions into a data‐driven era where the meaningful interpretation of large data sets is a limiting factor.

## 1 INTRODUCTION: SHAPE IS DATA AND DATA IS SHAPE

Shape is foundational to biology. Observing and documenting shape has fueled biological understanding, and from this perspective, it is also a type of data. At a glance, illustrations can reveal insights into relatedness and development. Ernst Haeckel's *Kunstformen der Natur* focuses on symmetry, structure, and pattern revealing differences and similarities in form throughout life. Shape can be documented with technology. The cyanotypes of Anna Atkins, a pioneer in photography, capture the exquisite branching patterns of algae while the microscopic renderings of Santiago Ramón y Cajal exposed the hitherto unknown realms of arborization within the brain. These glimpses into hidden realms rely on a shared ability to recognize patterns in experimental data: to look at a picture, see shape and form, and to extract meaning and latent information.

**FIGURE 1:**
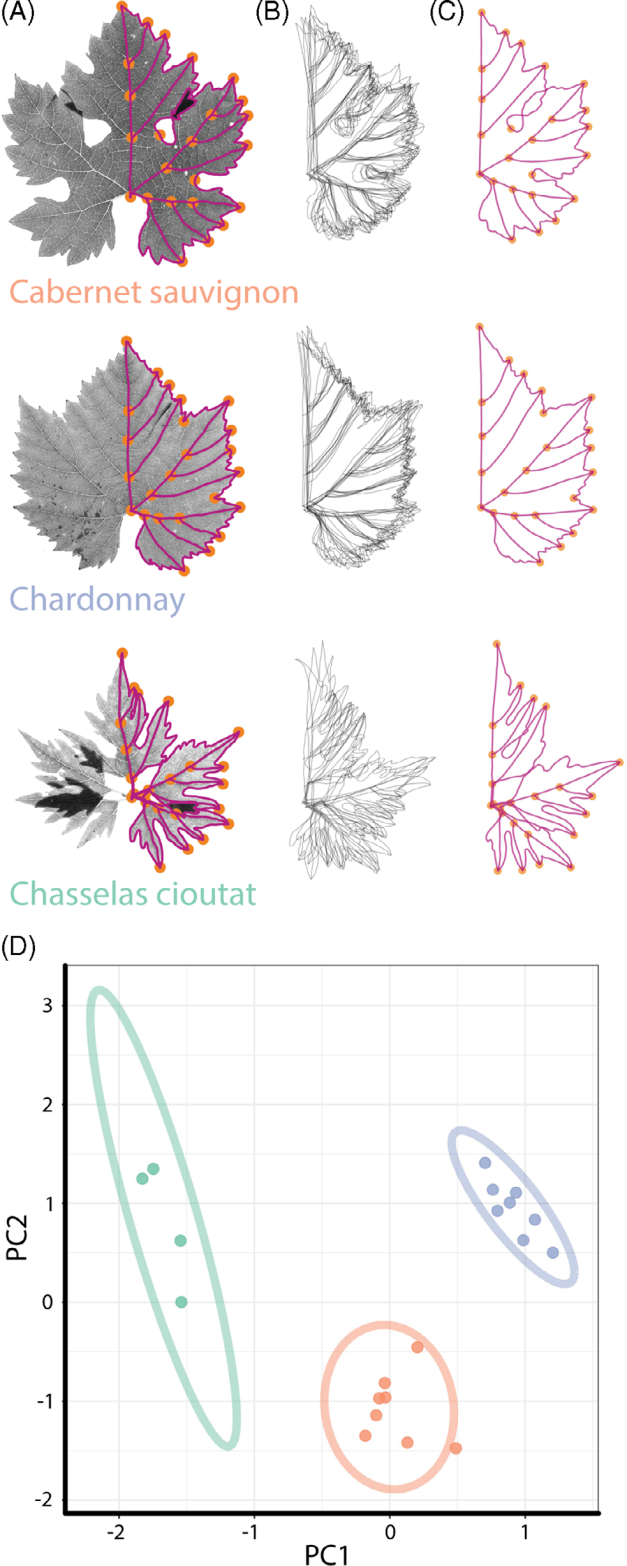
An example of geometric morphometrics. A, 24 landmarks (orange dots) and pseudo‐landmarks (6000 evenly spaced vertices between landmarks, magenta dots) on grapevine leaves of Cabernet sauvignon (orange), Chardonnay (blue), and Chasselas cioutat (green) varieties. Every grapevine leaf has five major veins, allowing corresponding landmarks to be placed throughout every leaf. B, Corresponding vertices allow replicates to be superimposed on each other, and C, mean leaves calculated using Procrustean methods that translate, rotate, reflect, and scale. D, A principal component analysis (PCA) and other statistics can be performed on the Procrustes‐adjusted vertices (95% confidence ellipses for each variety are shown)

Beyond observation and documentation, we can measure shapes, just as we analyze data. D'Arcy Thompson in his *On Growth and Form* used sheer mapping to linearly transform biological shapes between each other and described allometry, the relative growth of parts of an organism to the whole. Geometric morphometrics allows us to define distances between shapes by quantifying similarity and difference using landmarks, that is, sets of corresponding Cartesian coordinates^1^ (Figure 1). Sets of landmarks can be superimposed by translating, rotating, scaling, and reflecting to minimize the overall distance of shapes to each other.^2^ The process of superimposing sets of coordinates, known as Procrustes analysis, allows a metric space—the overall distance measuring the similarity of any pair of shapes—to be calculated and statistics performed. In the absence of a set of corresponding coordinates, the outline of a shape can be measured using Fourier analysis.^3^ A Fourier series is a summation of sine waves that approximate a complex wave. The discrete movements from pixel‐to‐pixel while traversing the closed contour (outline) of a shape can be analyzed using Fourier‐based approaches, describing the shape as a harmonic series, a summation of wave‐like elliptical contributions to the overall shape.^4^ Both geometric and Fourier‐based approaches quantify shape, exposing genetic, developmental, evolutionary, and environmental forces that sculpt the organismal form.^5^

Rarely do a finite set of landmarks capture the entirety of form that we see with our eyes, and often shapes lack corresponding points to define landmarks. A Fourier decomposition of a closed contour accurately recapitulates the outline of a shape, but how do we define features beyond silhouettes: pattern, texture, structure, and architecture? There are multitudes of shapes that defy definition using coordinates or outlines. Branching architectures—vasculature, trees, hyphae—not only abound in nature, but are the basis of abstract shapes, such as evolutionary trees.^6^ Networks (or in mathematical terms, *graphs*) are another abstract shape that can represent gene regulation, protein interactions, or metabolism in biology. Typically, we focus on individual nodes (or vertices, such as genes, proteins, or metabolites) but we could also analyze the overall shape of such a network (graph). We lack methods in biology to comprehensively describe the abundance of forms around us, from the molecular to organismal levels. We cannot extract the information we see with our eyes; there is a gulf between the biological information we know exists and the amount we can quantify.

Starting with a set of vertices, David Kendall in his *Shape manifolds, Procrustean metrics, and complex projective spaces* ^7^ describes a theory of shape upon which geometric morphometrics is built:

As topologists already have a theory of ‘shape’, I must apologize for using the word again with an entirely different meaning. In this paper ‘shape’ is used in the vulgar sense, and means what one would normally expect it to mean.

We return to sets of vertices and rather than describe shape in the vulgar sense, we focus on topology. Starting with a metric space, that is, data in which the distance of every point to every other is known, topological data analysis (TDA) allows the shape and structure of data to be measured.^8^ Data with distance can be points, pixels, or voxels; atoms, amino acids, or biomolecules; nuclei, cells, or organisms; or genetic and correlative distances. Not only can we use TDA to extract data from the shape, but also inherently TDA assumes that data have a shape. The concept of shape is only limited by the nature of the underlying data, and when considered in the abstract sense, the ability to measure shape becomes a powerful data analysis tool that can be applied to virtually any data set.

Here, we provide a middle ground between mathematics and biology: for mathematicians, a review of ways TDA has been successfully used to study biology and for biologists, an accessible introduction to topological thinking. We begin with examples from structural biology, evolution, cellular architecture, and neurobiology that lend themselves to simple but powerful TDA representations. We then examine shapes, focusing on the outlines of leaves, and the use of Euler characteristic curves as convenient topological signatures that enable statistical analyses. Next, we highlight ways that TDA can measure branching architecture and the use of bottleneck distance to calculate the overall topological similarity between objects. We end with a discussion about future trends in TDA: measuring dynamic shapes and time series as well as using topology to convert data to graphs representing its structure.

## 2 TDA: A PRIMER

### 2.1 Vietoris‐rips complex

Topology is the branch of mathematics concerned with mathematical properties that are preserved under continuous transformations. With some mathematical framework, described below, topology offers powerful tools that can precisely describe the overall shape and structure of the data encoded by a given network. Informally, we can think of the topology of a network as the collection of its features that remain unchanged whenever the data “varies smoothly.” For example, scaling, centering, translation, and rotation are all smooth operations that do not alter the topology (ie, the core shape) of our data. However, partitioning, merging, and attaching are not smooth operations and may significantly alter topology.

**FIGURE 2:**
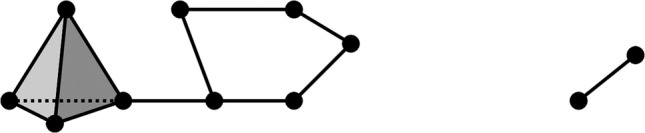
An example of a complex. It has two connected components, one loop, and one void

In a mathematical context, networks are referred to as graphs. Nodes or points are referred to as vertices, while links between nodes as edges. We can generalize the idea of graphs by adding triangles that link edges or even tetrahedrons that link triangles. More formally, we can think of our data as composed of different building blocks, called simplices. Vertices, edges, and triangles are zero‐, one‐, and two‐dimensional simplices, respectively. A collection of multiple simplices makes a simplicial complex, or complex, for short. For example, in Figure 2 we have a complex made of vertices, edges, and triangles.

We can describe the topology of a complex based on the number of its connected components, loops, and voids. For example, in Figure 2 we can see two distinct, separate pieces, each of them being a connected component. We see that five edges in the left component form the frame of a pentagon. We say then that these five edges form a loop. Also on the left, we see a collection of four triangular faces that form a tetrahedron. We can assume that this tetrahedron is hollow so that the complex contains a void.

Many times, our data or network cannot be immediately thought of as a complex. However, we can generate a complex based on a collection of data points and a notion of similarity or distance between these points. Formally, a collection of individual points and positive distances between every pair of points is referred to as a metric space. The Vietoris‐Rips (VR) complex is a versatile method to define a complex from a network. The VR complex starts with data in a metric space and a fixed nonnegative parameter *r*, often referred to as a radius. If two vertices are close enough, that is, the distance between them is less than *r*, then the VR complex will have an edge between those two vertices. Similarly, if there are three vertices close enough, that is, the distance between every pair of them is less than *r*, then the VR complex will have a triangle between those three vertices. Following these two rules, every time we have a triangle, we also have the three edges that make the frame or border of such a triangle. Conversely, every time a trio of vertices form a triangular frame, the VR complex will also contain the corresponding triangle.

### 2.2 Walking through an example

Notice that the same data and metric can produce different VR complexes by using a different parameter *r* each time. We can consider a sequence of increasing radii and its corresponding sequence of VR complexes. First, observe that the distance between any two different data points is always a positive quantity. If we start with *r* = 0, then the corresponding VR complex will consist solely of separate vertices, one for each data point. As the radius *r* increases, the corresponding VR complex will now have edges that link the pairs of vertices that are close to each other. If *r* keeps increasing, we may then have triangles that link trios of close vertices.

**FIGURE 3:**
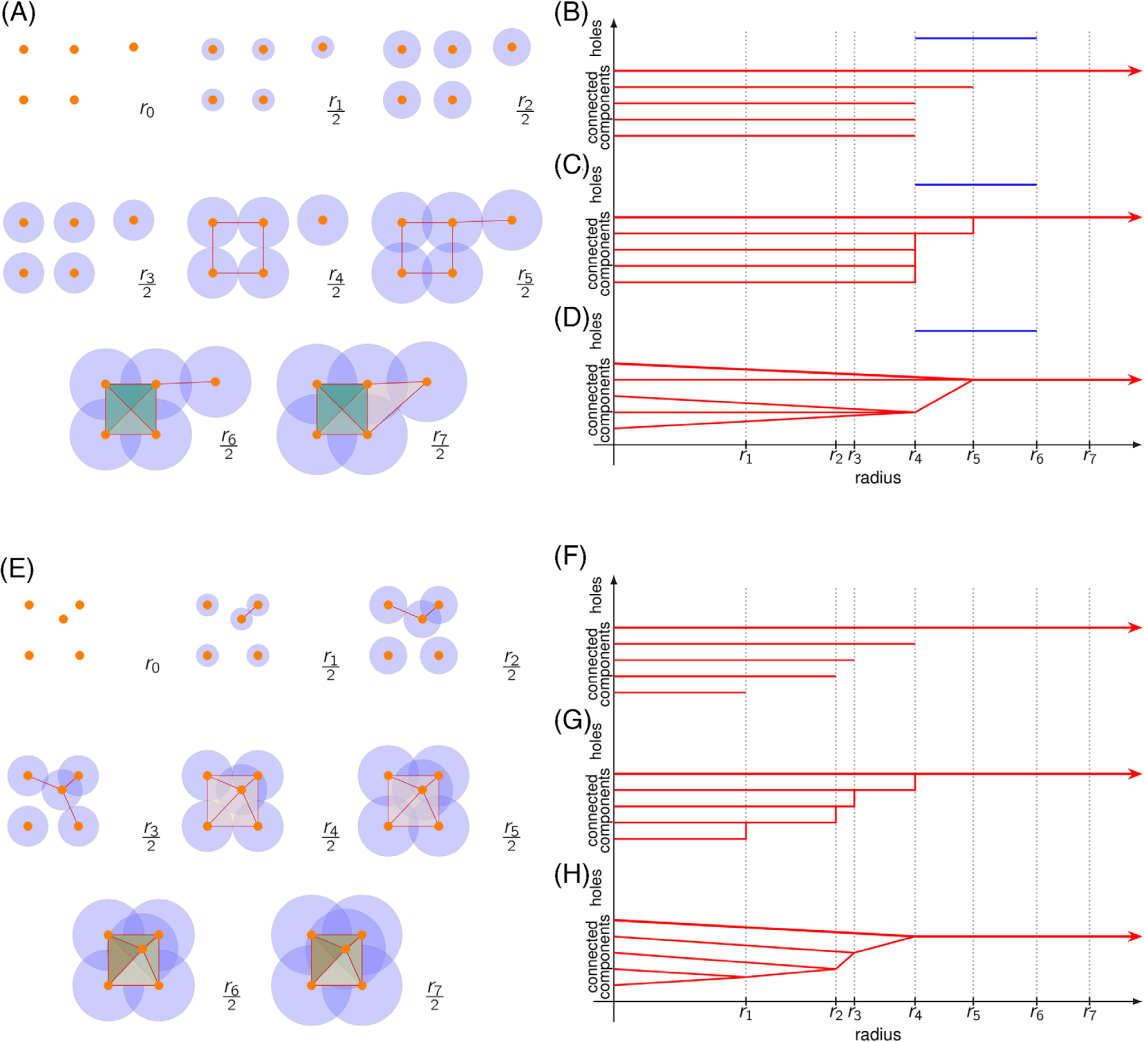
An example of two different Vietoris‐Rips complexes with resulting persistence barcodes. A, Evolution of a VR complex with five vertices as Euclidean distance increases. B, Persistence barcode corresponding to topological changes in the previous VR complex. C, Alternative visualization of the persistence of barcode B as a dendrogram. D, Alternative visualization of the persistence of barcode B as a tree. E, Moving one vertex in A yields a different VR complex as Euclidean distance increases. F, Persistence barcode corresponding to topological changes in the previous complex E. G, Alternative visualization of the persistence barcode F as a dendrogram. H, Alternative visualization of the persistence barcode F as a tree

For example, consider the five data points in Figure 3A, which we can take as vertices of a complex. The distance between the points will be simply the Euclidean distance. Consider seven different positive radii. For the first three radii, the shape remains the same: just five separate components. Suddenly, as soon as we increase the radius a fourth time, four pairs of vertices are finally close to each other so we draw edges between them to form a square. There is also a fifth vertex that remains isolated, as it is still distant from the rest. When the radius increases a fifth time, the isolated vertex is finally close enough to one of the square vertices. We draw one more edge at this point. The radius increases a sixth time so that the pair of diagonal vertices in the square is close enough. We then draw the diagonals of the square, which also draws the four possible triangles in the square. The radius finally increases a seventh time, so that the fifth vertex is closer to another vertex in the square. We then add an edge and a triangle including this fifth vertex. As the radius keeps increasing beyond this, the overall shape of the VR complex will not have any significant changes: it will always remain a single component with no holes.

### 2.3 Representing persistent features

All the observations described above can be summarized using two topological features: connected components and holes. For connected components, we need to keep track of which snapshot each connected component appeared (was born) and in which snapshot two separate components merged (died). Similarly, we can keep track of when each hole is formed (born), and when it is filled (dies). These life spans of topological birth and death can be drawn as life bars, the length of which indicates for how long a component persisted before it merged or how long a hole persisted before being filled. Putting all the bars together, we obtain a *persistence barcode*, in which each bar corresponds to a topological feature and the horizontal axis indicates at which radius value these features are born and die. Note that the vertical order of these life bars is irrelevant.

**FIGURE 4:**
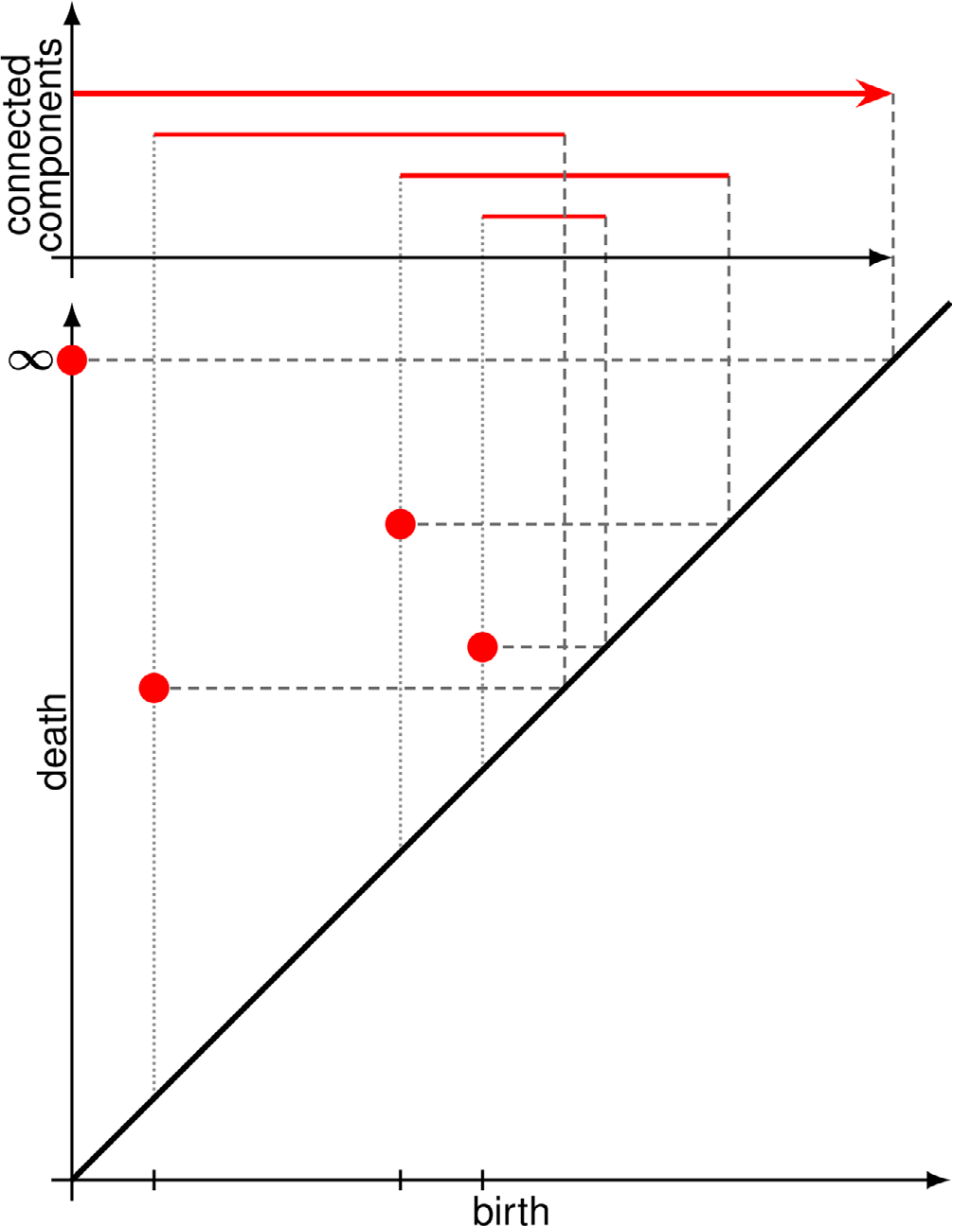
Translating a persistence barcode into a persistence diagram. Birth and death times in the persistence barcode are interpreted as x‐y coordinates on a death‐vs‐birth plane. This planar display is referred to as a persistence diagram

For the persistence barcode in Figure 3B, we observe that we start with five different vertices, all of which remain separate (the components persist) until the fourth radius. By the fourth radius, we only have two connected components: one square and one distant vertex. We also observe the birth of the hole in the square (indicated in blue). By the fifth radius, the distant vertex has merged with the square so we have only one connected component. By the sixth radius, we observe that the square hole has been filled with triangles. From this point onwards, as radius keeps increasing, our VR complex will be essentially a single connected component with no holes. We say then this component dies at infinity and it is represented by the continuing red arrow. We can alternatively display the persistence of components and holes as a dendrogram (Figure 3C) or a tree (Figure 3D), keeping track of which components merge. A particularly useful display of persistence barcodes are persistence diagrams, as illustrated in Figure 4. Simply, the birth start point and death endpoint of a bar in a persistence barcode are transformed as x‐y coordinates in a death‐vs‐birth plane in a persistence diagram. Persistence diagrams have a convenient visual and mathematical representation which has allowed further theoretical developments in TDA.

Barcodes are a useful way to illustrate and summarize prominent topological features, such as the distant fifth vertex or the hole enclosed by a square. Consider now “obstructing” this hole by moving the distant fifth vertex inside the square, as in Figure 3E. We observe in Figure 3F‐H a different persistence barcode, dendrogram, and tree, respectively. The barcode now shows that all five vertices merge into a single connected component at earlier stages compared to Figure 3A. Also notice that we have now filled the square's hole, so that the barcode in Figure 3F registers no holes, unlike Figure 3B.

### 2.4 Filters: Beyond spatial distances

As mentioned before, the VR complex is constructed from a set of vertices and a sense of distance or similarity between these. Sometimes, we refer to such a measure of similarity as a filter function since changing the maximum value of this function filters when the edges between vertices are observed. For example, in Figure 3A and E, our filter function was the Euclidean distance between vertices. Given a filter function, we can consider a series of snapshots, wherein each snapshot, we consider larger and larger filter values, called thresholds. Going back to Figure 3A and E, each snapshot considers increasing radius lengths around each vertex. In this case, we say that our collection of data points have been filtered by Euclidean distance with six thresholds. Notice that if we increase the number of thresholds, we may be able to capture finer topological changes which may, in turn, produce richer persistence barcodes.

**FIGURE 5:**
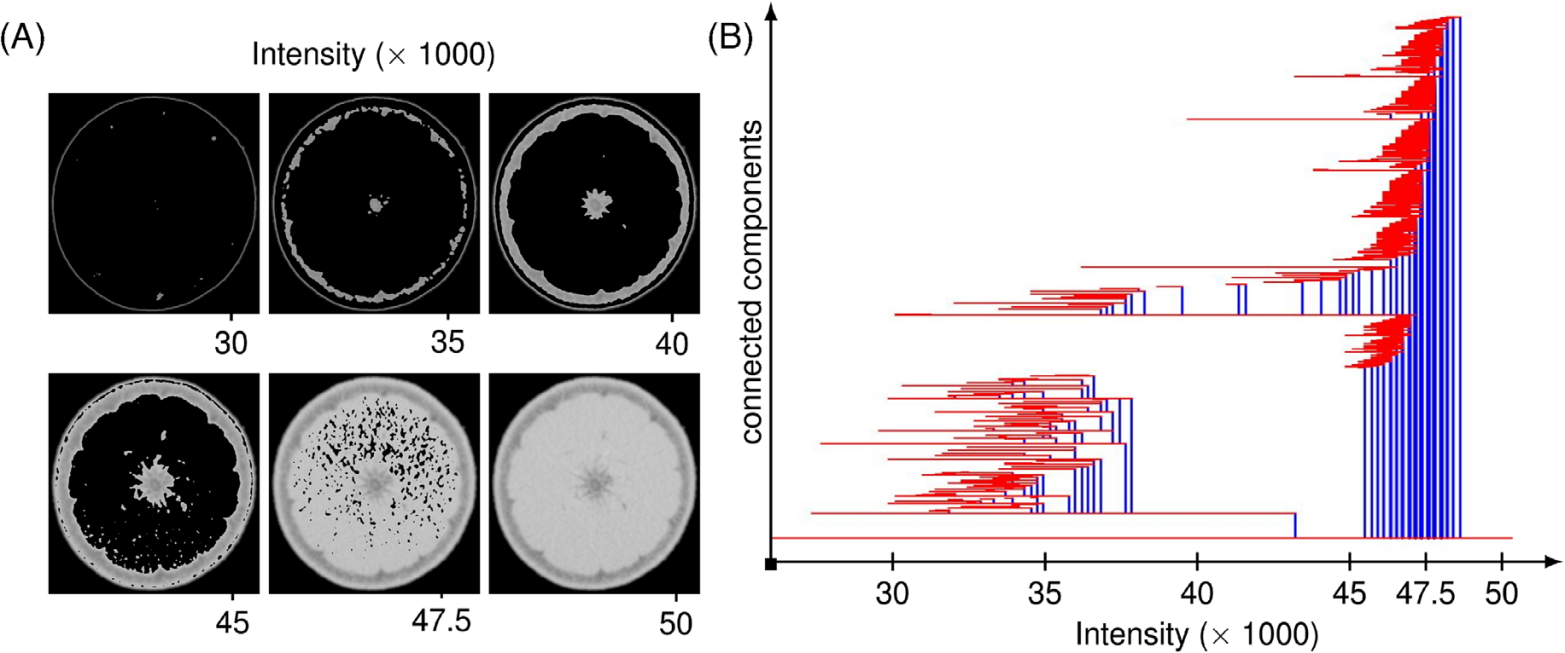
An example of a persistence barcode. A, Snapshots of an X‐ray CT image of an orange. Only the pixels with intensity lower than indicated are displayed. B, Persistence barcode of connected components of such an image. Observe that the barcode distinguishes the existence of exocarp, rind, and pith as separate components at lower intensities

Filter functions are extremely flexible and we can use more than spatial distance. For example, consider a gray scale image. We will consider each pixel a separate vertex and use an intensity filter, resulting in the distance between two pixels simply being the difference of their intensities. We can then consider each possible intensity value as a threshold. Figure 5 shows the persistence barcode of connected components from an X‐ray computed tomography (CT) scan of an orange where we consider more than 50 000 threshold values. We can look more carefully at some select snapshots in Figure 5A. In each snapshot, we only display the voxels (vertices) whose intensity value is less than the value of the threshold. At 30 000, we only observe the contour of the exocarp with some separate bits of rind. At 35 000, more bits (connected components) of rind appears, and some of these rind bits merge into each other. Additionally, we observe the appearance of the pith. By 40 000, we have three clear separate connected components, namely exocarp, rind, and pith. By 45 000, the rind and the exocarp have merged while numerous bits of endocarp have appeared. By 50 000, the appearance of the endocarp has merged the pith to the exocarp, yielding a single connected orange.

## 3 APPLIED TOPOLOGY: EXAMPLES FROM STRUCTURAL BIOLOGY, EVOLUTION, CELLULAR ARCHITECTURE, AND NEURAL NETWORKS

**FIGURE 6:**
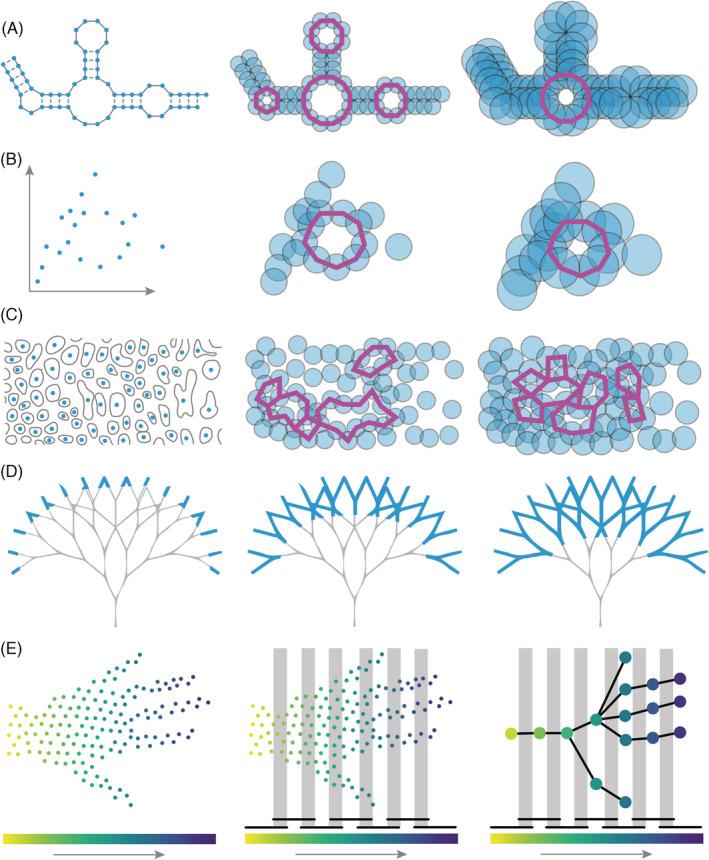
Applications of topological data analysis (TDA) to biology. A, Structural biology. A diagram of RNA secondary structure (left; solid lines covalent bonds, dashed lines hydrogen bonds). Increasing radii of vertices (middle, right; blue points) are used to visualize filtration on Euclidean distance. As radii merge, connected components die. Purple lines indicate the formation of loops that eventually fill in as the radius threshold increases. B, Evolution. A plot showing the genetic distance of samples (left). As radius threshold value increases (middle, right) the birth and death of connected components (blue) represent vertical evolution (a tree) while that of loops (purple) horizontal evolution events (such as hybridization, gene transfer, or recombination; modified from Reference 13). C, Cellular architecture. Modification of a part of the original Gleason guide to prostate cancer changes in cellular architecture (left). Nuclei (blue) increase in radius (middle, right) and connected components (blue) and loops (purple) are born and die. D, Branching architecture. A theoretical tree where the filter is the geodesic distance to the base (blue). Branching tips are separate connected components that merge as the filter progresses to the base of the tree (left to right). E, Mapper. Point cloud of a hand where the filter is the axes from the wrist to fingertips (left). Cover intervals (bars on top of the color scale) and their overlap (gray bars) divide points into bins (middle). Points that cluster together over each cluster are assigned to a vertex, and if the points are shared between clusters in an overlap, then they are assigned to an edge connecting the corresponding vertices (modified from Reference 42)

The VR complex framework introduced above, filtering on the Euclidean distance between data points, can be used to study a wide range of complex phenomena in biology. A metric space might be the 3D coordinates of atoms in a biomolecule like a protein or folded RNA, could represent species or virus variants separated from each other by genetic distance, might be defined by the nuclei of cells in a cross‐section of tissue, or be a correlation network of neural activity. Below, we provide examples where the VR complex has successfully been applied to structural biology, evolution, cellular architecture, and neural networks (Figure 6A‐C).

### 3.1 Structural biology

This prototypical example of TDA—a metric space consisting of points where the filter is Euclidean distance—can be extended to biomolecules, where the points are atoms or residues (Figure 6A). Proteins are comprised of a linear polymer of amino acids. The primary structure of a protein is the sequence of amino acids. The polypeptide chain of a protein folds upon itself, stabilized by interactions between amino acids, first forming a secondary structure (local structures such as alpha helices and beta sheets formed by hydrogen bonds in the peptide backbone) followed by the tertiary structure. The overall 3D structure of a protein is determined by the interactions of amino acid side chains within the protein. This overall structure, or conformation, of a protein is the basis of protein function: metabolism, transport, signaling, structure, and movement, among many others. The conformation of a protein can change depending upon binding ligands, signaling, or the chemical environment.

Kovacev‐Nikolic et al.^9^ use TDA to distinguish the open and closed conformations of maltose‐binding protein (MBP). Each of the 370 amino acids of MBP is treated as a vertex, its 3D coordinates reflecting the spatial location of the residue. Euclidean distance is used to create a filtered VR complex for each protein studied. As the VR complex evolves, persistence barcodes record the birth and death of connected components, loops, and voids, which within the context of the tortuous folding of a protein backbone, yield complex topological signatures unique to distinct conformations. These persistence barcodes are transformed into *persistence landscapes* ^10^ that allow statistics, hypothesis testing, and machine learning to be applied to differentiate the shapes captured by topological signatures. The authors successfully differentiate open‐ and closed‐conformation states of MBP. They also note that the active site residues (the amino acids responsible for ligand binding) lie at the edge of the most persistent loop of the VR complex, indicating that TDA is sensitive to the relationship between structure and function. Beyond structure, electrostatic and other chemical properties of atoms can be incorporated into topological signatures that, when analyzed using machine learning methods, can predict protein‐ligand binding affinities.^11^, ^12^

### 3.2 Evolution

Evolution is typically depicted as a tree, which in mathematical terms is an acyclic graph (a graph with no loops). Each node and its descendant branches represent a common ancestor of a taxonomic group and its members as a hierarchy of similarity or relatedness. Evolutionary trees depict *vertical evolution*, random mutations that accumulate within a specific lineage that lead to phenotypic changes. However, genetic material can be exchanged between lineages as well. This process, known as *horizontal evolution*, is depicted as a reticulate graph (with loops), in which genetic information is exchanged by recombination, hybridization, horizontal gene transfer, or viral reassortment. Extensive phylogenetic theory models vertical evolutionary processes using trees, but the study of horizontal evolution is often limited to detecting reticulate phylogenetic events, and a theory unifying vertical and horizontal evolution has remained elusive.

Chan et al.^13^ reconsider evolution from the perspective of topology. Using influenza as an example, they begin by considering that every sample has a genetic distance to every other, a metric space. From this genetic space, they construct a VR complex, just as in the previous examples (Figure 6B). The resulting persistence barcode for connected components can be converted into a dendrogram, which is the phylogenetic tree that biologists are accustomed to. Influenza viruses extensively exchange genetic material in a process known as reassortment. If a persistence barcode is generated for loops, then this represents a topological signature of horizontal evolution. For example, a lower bound of recombination rate can be calculated from the number of loops (recombination or reassortment events) for a given time frame (in this example, the filter of genetic distance which can be calibrated to time). In higher dimensional spaces, voids can be detected, and the authors show that persistence barcodes for voids detect more complicated reassortment events, such as the triple reassortment that gave rise to the 2013 avian influenza outbreak and complex reassortment events within HIV. Using loops as an estimator of recombination rate has been extended to large‐scale genomic analysis^14^ and humans,^15^ expanded upon by evaluating different topological features,^16^ applied to coalescent theory to estimate ancestral recombination events,^17^ and used to study lateral gene transfer of protein families and its implications for the evolution of antibiotic resistance.^18^

### 3.3 Cellular architecture

Tissues are comprised of cells, the organization of which is determined by cell division, differentiation, growth, movement, migration, and death. Within a tissue, each cell takes up a finite volume, often in close contact with neighbors. When a tissue is finely cross‐sectioned and microscopically examined, a tessellated array of cells emerges: an aggregated mixture of parenchyma, stroma, and glands (Figure 6C). Staining can differentiate nuclei, cytoplasm, and extracellular matrix. To a trained eye, these complex patterns can indicate disease or abnormalities, but the process takes time and is subjective. The emergent organization of cells reflects developmental processes as well. TDA provides an objective way to classify these patterns, potentially removing the subjectivity of histopathological diagnosis enabling a rigorous way to define cellular anatomy.

Lawson et al.^19^, ^20^ explore the cellular architecture of prostate cancer. The Gleason grading system is a one to five scale that is a powerful prognostic indicator based on increasingly neoplastic tissue organization of the prostate: a uniform cellular architecture becomes disrupted forming glands that eventually form solid cell types. Tissue sections are stained with blue‐purple hematoxylin and pink eosin which indicate nuclei and cytoplasm/extracellular matrix, respectively. The authors use these stains to isolate cell nuclei from surrounding structures (Figure 6C). They then use thresholding as a filter on histological images of prostate cancer to create binary images, where connected components and loops are recorded as persistence barcodes. Creating vectors of the most persistent features, they use a variety of statistical techniques including principal component analysis (PCA), hierarchical clustering, and t‐distributed stochastic neighbor embedding (t‐SNE) to successfully classify images according to the Gleason grading system. The strategy of reducing cells to data points of a VR complex to classify cellular architecture works for predicting epithelial organization from cell centroids^21^ and in other cancers as well.^22^, ^23^

### 3.4 Neural networks

The complex architecture of neurons and their numerous connections in the brain, formally referred to as the connectome, is of particular interest. The architecture of the brain, its activity, and connectivity, is usually presented as a square pair‐wise correlation matrix where each row (and column) represents different neurons or encoding brain regions when the subject is performing a fixed task. Usually, negative correlations are treated as zero, and the rest of matrix entries are thresholded so that only neurons or regions with strong correlation are considered connected. We can then consider a metric space where the points are different neurons, anatomical regions of interest, or imaging voxels. The distance between these points is given by the correlation between them (or one minus correlation to be mathematically consistent). With this setup, it is possible to produce VR complexes and persistence diagrams that summarize the brain network model.

Observing the change in the number of connected components, Lee et al.^24^ differentiate the abnormal glucose metabolism associated with neuronal activity between attention‐deficit hyperactivity disorder (ADHD) children, autism spectrum disorder (ASD) children, and pediatric control subjects. On the other hand, by keeping track of persistent loops, Petri et al.^25^ distinguish effects of psilocybin on human brain functional patterns, while Ibañez‐Marcelo et al.^26^ highlight that mental imagery shares the same neurophysiological bases with perceptual and motor experience. TDA has also revealed previously ignored anatomical loops and voids in the connectome, which might explain both spatial and nonspatial behaviors both in mice^27^ and humans.^28^

## 4 SHAPE, TEXTURE, AND THE EULER CHARACTERISTIC CURVE

The examples above rely on point‐based representations of biological data to which a filtered VR complex on Euclidean (or genetic) distance produces zero‐dimensional (connected components) or one‐dimensional (loops) persistence barcodes. The persistence of the barcodes, representing prominent topological features, is the focus of analysis. However, the concept of the filter can be extended to any real values that can be associated with structures: for instance, different choices of metric space between data points, or even using filter functions based on the data points instead of the edges between the data points. For the same data, many filters might be applied, yielding a new lens to reveal different facets of shape. Persistence barcodes can always be calculated, but there are other ways to record topological signatures as well.

Below, we first describe the Euler characteristic curve (ECC) as a convenient complement to persistence barcodes to capture topological signatures that can be used with traditional statistical methods. We then describe TDA frameworks to measure shape (in the traditional sense of a closed contour) focusing on leaf outlines and the usefulness of ECCs to measure genetic and environmental effects that determine phenotype.

### 4.1 ECC

The Euler characteristic, often denoted by the Greek letter *χ*, was originally defined by the equation:

$$\chi =\#\left(\text{Vertices}\right)-\#\left(\text{Edges}\right)+\#\left(\text{Faces}\right).$$

The Euler characteristic is the first example of a topological invariant; that is, a quantity that can be calculated and returns the same value on many different representations of the same topological shape. For convex polyhedra (eg, the Platonic solids), the Euler characteristic always equals two since all platonic solids are topologically spheres. For example, a tetrahedron has 4 vertices, 6 edges, and 4 faces (4 − 6 + 4 = 2); a cube has 8 vertices, 12 edges, and six faces (8 − 12 + 6 = 2).

What is even more surprising is that this quantity can also be obtained by counting some intrinsic properties of a given shape. The Euler‐Poincaré formula establishes that the formula above is the same as:

$$\chi =\#\left(\text{Connected Components}\right)-\#\left(\text{Loops}\right)+\#\left(\text{Voids}\right).$$

So, since all convex polyhedra have one connected component and one void, the Euler characteristic is still seen to be 1 − 0 + 1 = 2. Then, of course, it is easier to see the value of the Euler characteristic for other structures. For example, a doughnut, mathematically known as a solid torus, has one connected component, one loop, and no voids so its Euler characteristic is 1 − 1 + 0 = 0.

If the Euler characteristic is applied to TDA, by keeping track of the number of building blocks of our simplicial complex, we can indirectly summarize its topological features.

**FIGURE 7:**
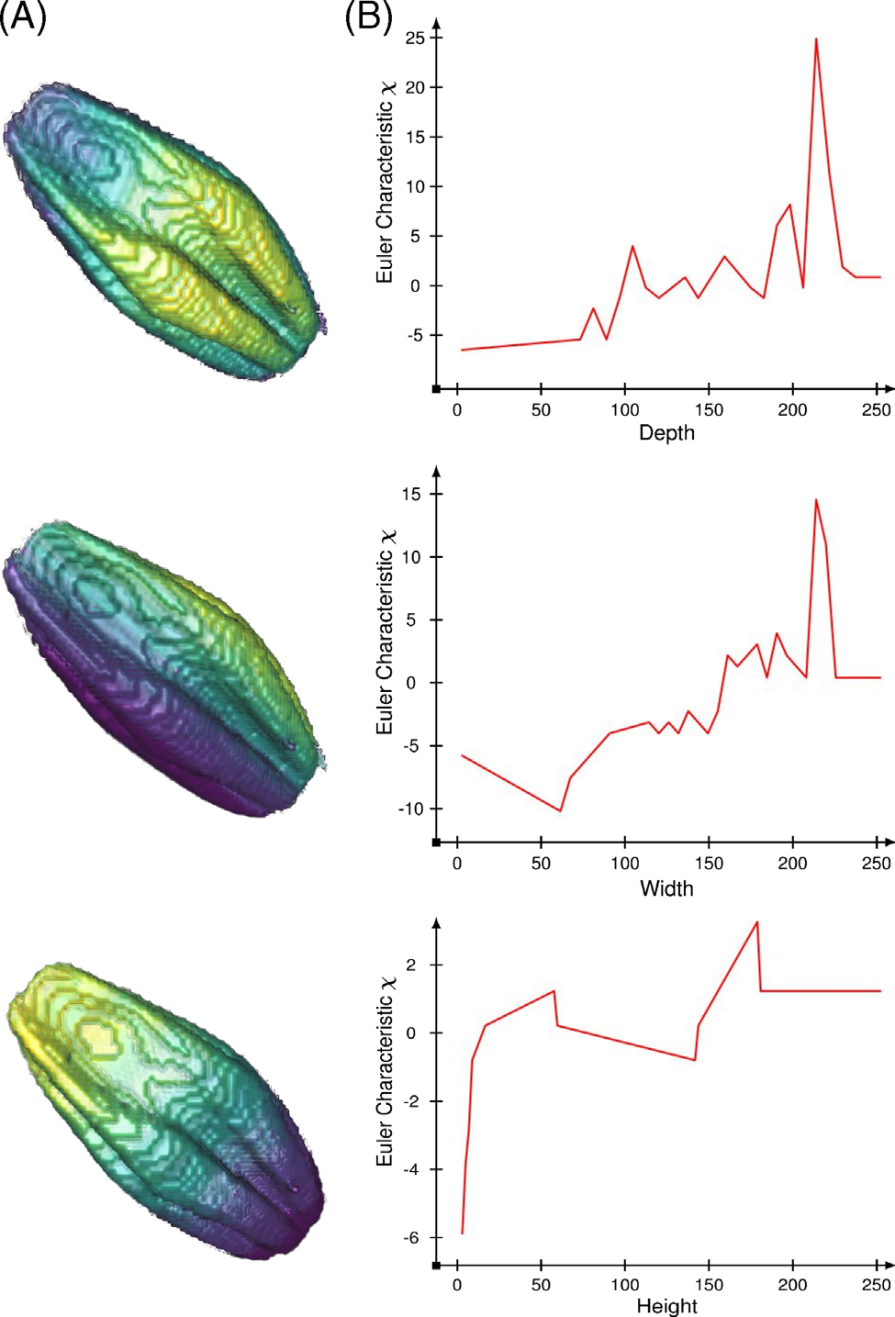
Three different Euler characteristic curves (ECCs) from three different filters. A, X‐ray CT scan of a barley seed. The symmetry of the seed encourages a filter by depth, width, and height values, that is, the three main axis directions with respect to the seed scan. Slicing the barley seed in different directions produce, B, different corresponding ECCs. Notice that the three curves end with Euler characteristic equal to one, which corresponds to the Euler characteristic of a solid sphere

Similar to persistence barcodes, given a data set, for each sample, we define vertices, a filter function, and many thresholds. For example, consider a 3D (voxel‐based) image of a barley seed (Figure 7A). Each voxel is a vertex in our simplicial complex. One type of filter we can apply is the 3D axes of the coordinate system, which is oriented with respect to the depth, width, and height of the seed. For each of these filters, the voxels take the real number value of their coordinate for the particular axis. We then choose many thresholds, or equivalently, we choose how many times to “slice” through the seed along the given axis. Each time we take a slice, we compute the Euler characteristic of the seed. We continue to add slices one‐by‐one and recalculate the Euler characteristic each time as we continue through the axis, which is the filter function. Adding all the slices together yields the original seed. Finally, we summarize our computation as an ECC (Figure 7B), where the x‐axis is the threshold while the y‐axis is the Euler characteristic of the complex at that particular threshold value.

Persistence barcodes tend to be notoriously expensive and difficult to compute since they must keep track of all the possible component merges and hole fillings for every threshold value. Most of the available software to compute persistence barcodes is incapable of handling truly large data sets effectively, especially when each sample consists of millions of vertices. Euler characteristic curves are a convenient way to summarize a topological signature of an object as a sequence of numbers, a curve, or a numerical vector. Computing and storing these vectors is quite efficient, and it is especially convenient since it allows us to perform standard statistical analysis techniques and test hypotheses about the shape of our data.

### 4.2 Shapes and textures

Sometimes leaves have corresponding coordinates, as in the case of grapevine where every leaf has five major veins and numerous landmark features^5^ (Figure 1). In these instances, geometric morphometrics is a powerful tool. Besides the base of the petiole and tip, though, most leaves do not have coordinates that correspond in a way that analysis by geometric morphometrics is possible. To compare the outlines of 182 707 leaves from 141 plant families and 75 sites throughout the world, Li et al.^29^ used TDA. The pixel outline of each leaf is treated as a point cloud. The filter applied to each pixel is a Gaussian density estimator, sensitive to the number of neighboring pixels around each pixel. Straighter edges of the leaf blade will have low‐density values while pixels in serrations, lobes, or other undulations will have higher values. The number of connected components is monitored and the respective ECCs are computed.

For so many leaves, an ECC curve serves as a succinct, computationally feasible topological signature that allows downstream statistical analyses. Li et al.^29^ are able to compute a morphospace for all leaves (which reveals not only the leaf shapes that exist, but those that do not, either because of developmental constraint or negative selection) and use ECCs to predict plant family and location. Others have used the same filter and ECCs to determine the genetic basis of leaf shape in apple^30^ and tomato^31^ as well as the genetic basis of cranberry shape.^32^ ECCs are sensitive enough to complex and subtle changes in shape to measure the effects of rootstock and climate on grapevine leaf shape.^33^ ECCs have also been used to measure the hairiness and shape of spikelets (arrangements of grass flowers)^34^ and patterns of vegetation from satellite imagery.^35^

## 5 BRANCHING ARCHITECTURES AND BOTTLENECK DISTANCES

### 5.1 Persistence diagrams

The Euler characteristic allows us to monitor a topological summary as a function of the filter we choose. The resulting curve enables statistical analyses. In some cases, we might not want a summary, though; we may want to keep track of each topological feature separately, as we do in a barcode. The bottleneck distance is a convenient way to determine the overall topological similarity of two barcodes with each other. If we compute the bottleneck distance of all barcodes to all other barcodes, we can determine the overall topological similarity of samples to each other, in which case statistical analyses can be performed. To understand the meaning of bottleneck distance, we need a better display of topological information than persistence barcodes. We thus turn to persistence diagrams.

As mentioned previously, each topological feature in the barcode has a birth and death time. Instead of representing a topological feature with a life bar as in persistence barcodes, we can simply represent it with a point in a plane; the x‐coordinate of this point is the birth time of the topological feature, while the y‐coordinate is its death time. All of our topological information is then displayed in a death‐vs‐birth plane, referred to as a persistence diagram. Certainly, a topological feature cannot die before it is born, so all our points will lie above the diagonal line. We also agree that the top of the plane will represent infinite time, for those features that persist until infinity.

Consider a very simple persistence barcode as shown in Figure 4. The birth and death times of each life bar are read as x‐y coordinates on the plane below. Observe that the barcode presents a component that persists until infinity. Thus, we define an “infinite death time” at the top of our diagram.

### 5.2 Bottleneck distance

For ease of exposition, we will describe bottleneck distance in terms of persistence diagrams rather than persistence barcodes as they are equivalent. Intuitively, the bottleneck distance between two diagrams measures how much change the first sample must undergo so that its resulting persistence diagram matches the diagram of the second sample. More formally, think of bottleneck distance as follows: we overlap the persistence diagrams of two samples, so both diagrams are actually on the same plane. Next, we are tasked to pair topological features between the diagrams. Every point from the first diagram must be either paired to an unmatched point from the second diagram or matched with the diagonal. Given a pairing, we define its score as the maximum distance between pairs. After considering all possible pairings, the bottleneck distance is defined as the minimum possible score.

**FIGURE 8:**
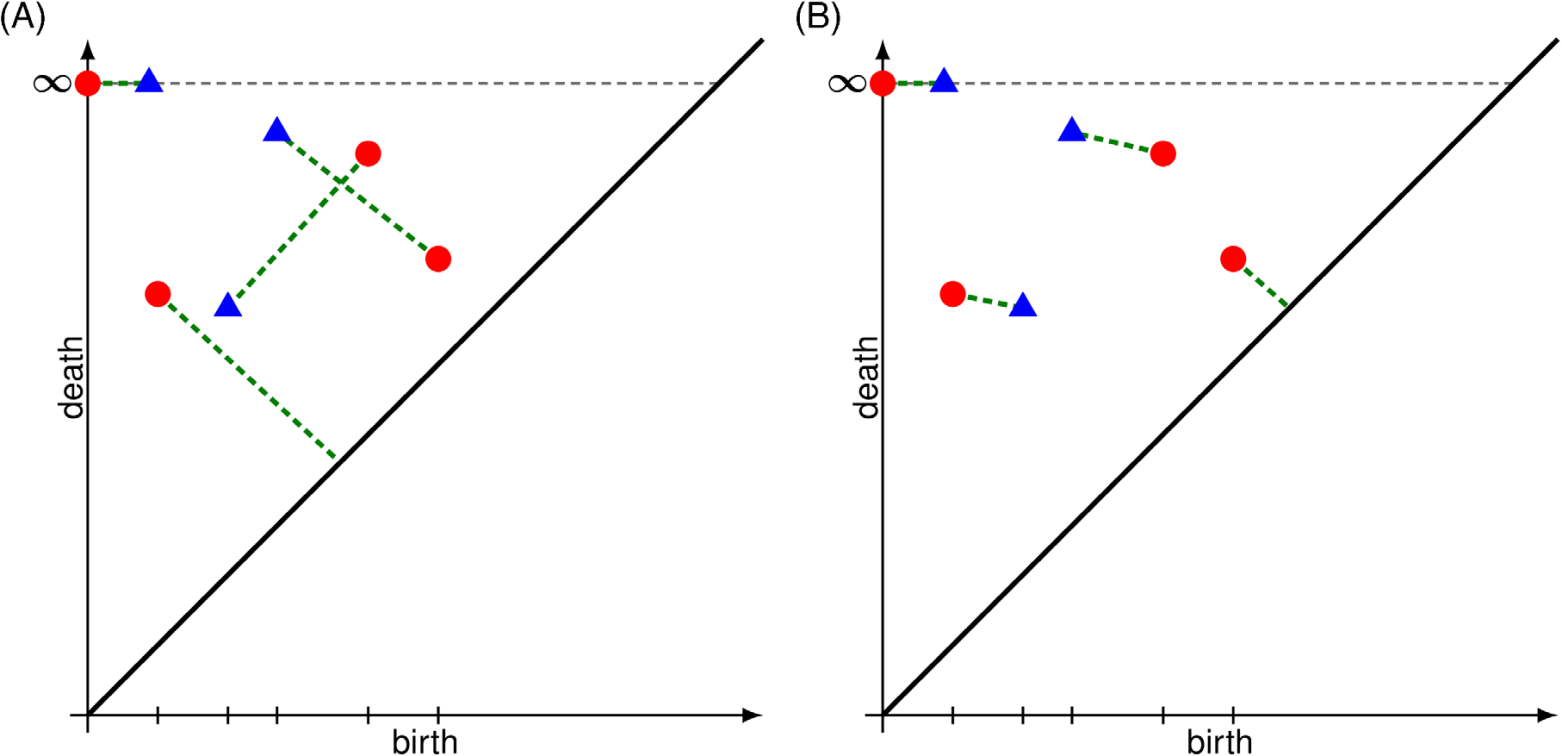
Computing the bottleneck distance between two persistence diagrams. A, A possible pairing of points is suggested. Observe that it produces a large maximum distance between pairs. B, An alternate pairing that yields a considerably smaller maximum distance between pairs

For example, consider the two different persistence diagrams drawn on top of each other in Figure 8, the first one is represented with red circles while the second with blue triangles. In Figure 8A we pair each triangle with another circle, taking care to match the infinite triangle with the infinite circle. Observe that one circle is matched to the diagonal. The score of this pairing is the length of the longest green, dashed line. A different pairing is considered in Figure 8B, which in turn produces a considerably smaller score as the green lines are all considerably shorter. After considering all possible pairings between diagrams, we realize that Figure 8B is optimal in the sense that it produces the smallest score. The bottleneck distance between these barcodes is then this minimum score.

### 5.3 Branching architectures

Branching architecture is one example where bottleneck distance is useful. Traditional morphometric approaches fail to measure branching, despite it being a common architectural motif throughout life.^6^ Li et al.^36^ measure the branching architecture of X‐ray Computed Tomography (CT) scans of grapevine rachises, the branching stem structure that remains after removing the berries from the cluster. The filter they choose is a geodesic distance of each voxel to the rachis base (Figure 6D). The geodesic distance is the shortest distance between two vertices on a surface, in this case, the grapevine rachis itself. Starting with those voxels with the furthest geodesic distance from the base and filtering towards those closest, if zero‐dimensional features are monitored, connected components at the tip of the branching structure are first born and then die as they merge at their parent node. Connected components continue to arise at branch tips and die at parent nodes in a hierarchical fashion. Each topological feature corresponds to a bar as in Figure 3, and the record of merging can create a dendrogram that recapitulates the branching. There are many filters sensitive to branching that have been used in both plants and other organisms.^37^, ^38^, ^39^, ^40^, ^41^

Branching is an instance where calculating bottleneck distance might be preferred to Euler characteristic curves because the topological features more directly correspond to the feature of interest (branches). Calculating the bottleneck distance of each grapevine rachis to the other^36^ creates a metric space from which samples can be hierarchically clustered based on morphology. Comparing morphological similarity to evolutionary history, rates of evolution along branches of the phylogenetic tree can be modeled. The morphological similarity matrix calculated from bottleneck distances can also be compared to traditional measurements (such as the number of branches, median branch length, and width, convex hull) and the ability to classify rachises from different species.

## 6 THE STRUCTURE OF DATA: MAPPER AND BIOLOGICAL NETWORKS

### 6.1 Mapper

Topological signatures and TDA outputs—barcodes, Euler characteristic curves, and bottleneck distances—measure the shape of data comprehensively but lack a correspondence to the original data. This is known as the inverse problem: from data, we can calculate a topological signature, but from a topological signature, we cannot resynthesize the original data. Biological data are noisy, and if the shape of the underlying structure in data could be visualized, individual data points that contribute to the overall shape of data could be isolated and studied in detail. For this reason, we now turn our attention to the mapper graph, which does provide some information in the reverse direction. By delimiting an underlying structure to our data and assigning correspondence of data points to this structure, complex and noisy data sets are simplified in a way similar to data reduction techniques.

**FIGURE 9:**
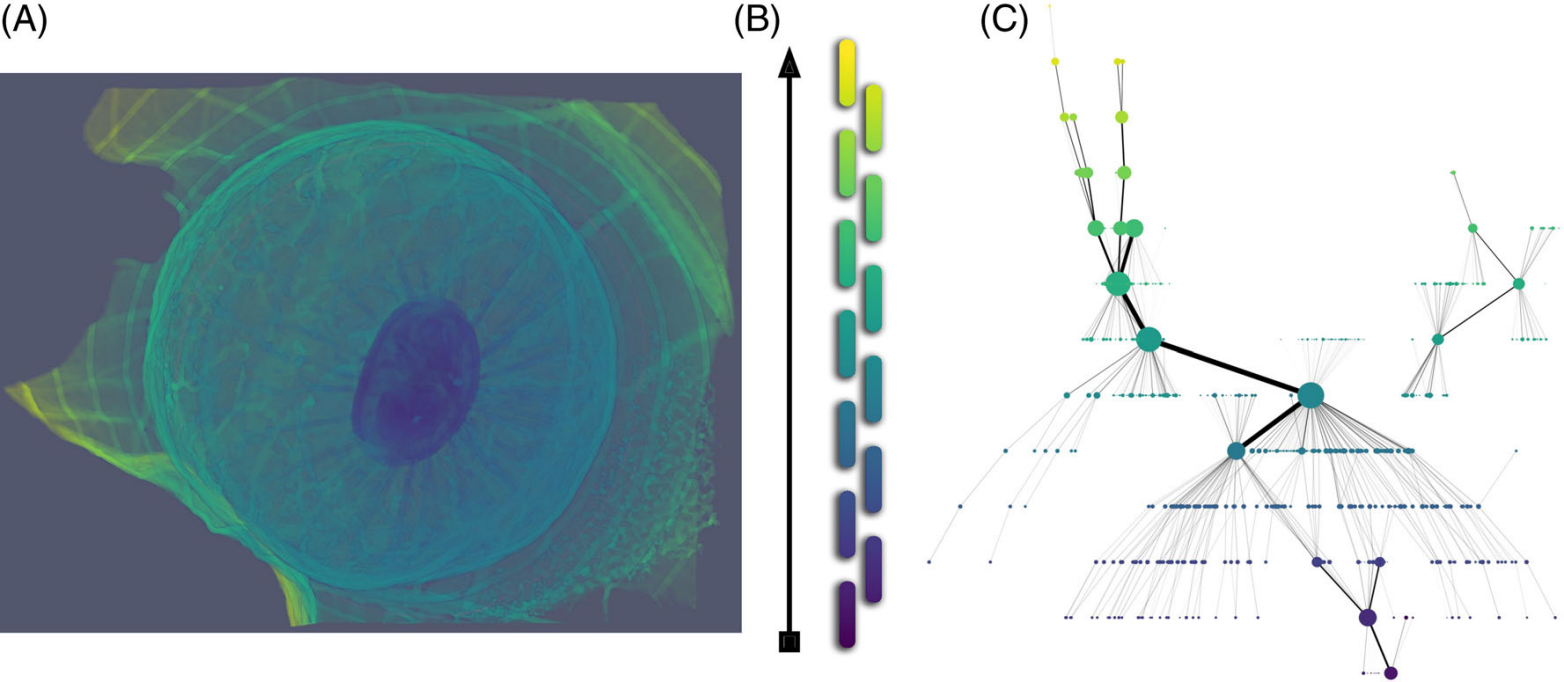
An example of mapper graphs. A, X‐Ray CT scan of a gall filtered by distance from the center. B, These filter values are projected to a real line. The real line is then covered by a collection of overlapping intervals. For each interval, we then form different clusters of voxels whose filter value is in such interval. These clusters then yield the vertices and edges of, C, a mapper graph. Formally, the vertices are connected components within a certain range of radius from the center and edges correspond to overlap. Size of vertices and edges corresponds to the size of the component or overlap

Mapper is a tool from TDA that skeletonizes and summarizes the shape and structure of data as a graph.^42^ The Mapper algorithm is comprised of three main steps. (a) We choose a filter function on the data (Figure 9A), this time associated with vertices rather than edges, and project all data points onto a line according to their filter function values (Figure 9B). (b) Next, we split the real line into a fixed number of bins called covers. Each cover is an interval over the filter and, additionally, there is an overlap between the covers. (c) Finally, we cluster the original data points in each of these bins to form graph vertices. Edges are drawn between nodes in the mapper graph if two clusters share some data points (Figure 9C).

Imagine a 3D point cloud of data shaped like your hand, where the filter function is the distance of each data point to your wrist^42^ (Figure 6E). If we created overlapping intervals, or covers, along this axis, then points at the fingertips would each form a vertex, and points towards the base of the fingers would form their vertices as well. Because there is overlap between the covers, then vertices along each finger, but not between fingers, would share points, and we would draw in edges between these groups of points that would recapitulate the structure of fingers. The most proximal finger vertices would converge with vertices representing the palm, as well as vertices of the thumb. In the case of a hand, it is easy to see how a mapper graph summarizes and recapitulates the structure of the actual data. When applied to real‐world data, such as volumetric images like an X‐ray CT scan, mapper graphs recapitulate shape in intuitive ways.^43^

Let us consider a voxel‐based X‐ray CT scan of a gall (Figure 9A), a swollen plant growth induced by an insect for its benefit. Each voxel is a data point that takes on the value of the filter function, which in this case is its distance from the center of the gall. The Mapper algorithm clusters the data into vertices based on their filter function value (Figure 9B), and if two vertices share some voxels between them (based on the cover intervals assigned and the physical location of the voxels), then they are connected by an edge. Bigger vertices in the mapper graph (Figure 9C) correspond to a larger number of clustered voxels. Thicker edges correspond to a larger number of voxels in the bin overlap. The color of the vertices corresponds to the average filter function values of its voxel members. At the bottom of the graph, we can see a purple cluster corresponding to the core of the gall. As we move up, we eventually find larger turquoise vertices corresponding to the outer layers of the gall. Notice the small vertices that stem from these large turquoise vertices which represent the vasculature of the gall. Finally, as we reach the top of the mapper graph, we find green and yellow vertices that represent the leaf. From this example, two important features of mapper can be seen: its ability to serve as a data reduction technique that summarizes the structure and the correspondence of the graph to the original data.

### 6.2 Biological networks

We have focused on Euclidean distances up until this point. However, just like genetic distances can be used to create metric spaces to study evolution, other distance metrics can be used to create graphs that can be studied with TDA as well. Nicolau et al.^44^ used mapper to identify breast cancer subtypes using gene expression microarray data. The filter they use decomposes their data into separate normal and disease components. The resulting mapper graph reveals three distinct arms that, upon subsequent analysis, reveal a distinct genetic subtype of tumors. The architecture of the mapper graph corresponds to disease progression and its vertices to the expression of genes linked to breast cancer subtypes. By choosing an appropriate filter, the mapper graph reveals a structure of the data that might have been missed otherwise and is linked to prognosis.

Mapper has also been used to reveal the underlying structure of cell lineages and development using single‐cell RNA‐Seq data. Single‐cell RNA‐Seq is a method that captures the gene expression profiles of individual cells. Dimension reduction techniques combined with knowledge of cell type‐specific markers can reveal the evolution of gene expression profiles during differentiation. Similar to the example above, Rizvi et al.^45^ use a filter related to their question of interest, which is based on first creating nodes of genes with high connectivity and then assigning a root node based on sampling time that corresponds to the undifferentiated state. The remainder of nodes are assigned values based on their distance from the root. Mapper is a powerful method to analyze a single‐cell RNA‐Seq data set of motor neurons differentiating from murine embryonic stem cells, as the resulting mapper graph reflects the process of differentiation itself.

## 7 A WORD OF STATISTICAL CAUTION

Most of the time, our data are subject to different kinds of errors and we must address the statistical robustness of our topological signals. One foundational result by Cohen‐Steiner et al.^46^ is the stability of persistence diagrams with respect to the bottleneck distance. Intuitively, this stability result implies that if all our data points wiggle only a little bit (possibly due to noise), then the resulting points in the persistence diagram will only wiggle a little bit as well. We must be careful with outliers though, as illustrated by Figure 3 since a single outlier can significantly alter our persistence diagram. Nonetheless, there has been many ideas to address this lack of robustness with respect to outliers, such as using a distance to measure^47^ or multi‐parameter persistence.^48^, ^49^ Intuitively, since an outlier is distant from every other point, it will lie in a low‐density region, so we then proceed to discard such regions.

It is worth to warn that the space of all possible persistence diagrams is a mathematically complicated space to work with. For instance, given a collection of persistence diagrams, there might not be a unique “mean diagram.”^50^ The space of persistence diagrams presents many difficulties to define *P*‐values, or confidence intervals, which are crucial in any statistical analysis. However, there has been a growing number of ideas and research to address such pitfalls, such as modifying the bottleneck distance to explicitly construct “mean diagrams”,^51^, ^52^ adapting randomized null hypothesis tests,^53^ or defining a confidence interval line along the diagonal of the diagrams.^54^ Other alternatives include transforming diagrams to a simpler and more sound space, as done with persistence landscapes,^10^ where the usual statistics, parameter estimation, and hypothesis testing can be carried out as usual.

Another caution to make is the interpretability of topological signatures. While summaries as persistence landscapes and ECCs are powerful when combined with machine learning techniques, it is hard to directly identify phenotypes from them. For instance, it is difficult to deduce the length, height and width of a seed‐based solely on the ECCs from Figure 7B. Turner et al.^55^ mathematically prove that the collection of all ECCs corresponding to all possible directions effectively summarizes all the morphological information for 3D and 2D shapes. Moreover, with such a collection we would be able to reconstruct the original object. Nonetheless, in practice, we cannot consider an infinite number of directions. A finite bound on the number of necessary directions for general 3D shapes has been proven,^56^, ^57^ although the idea of efficiently reconstructing large objects solely from ECCs remains elusive.

## 8 CONCLUSION

We have seen how given data, a summary of the topological shape and structure of the space can be computed. For instance, data could come as a metric space of any distance—whether Euclidean, geodesic, genetic, functional, or correlative—and we can return a VR complex and corresponding persistence barcode, which measure the shape of our data. By monitoring connected components, loops, or higher‐dimensional features, the barcode captures shape comprehensively, by monitoring the evolution of these features as a function of the filter. Such a framework has been used to measure the shapes of proteins, model evolution, and classify tissue architecture. The filter that we choose is arbitrary: it is merely a lens through which we can view relationships between our data points. The ability to choose a filter tailored to the hypothesis at hand is what confers the versatility of TDA to measure the shape of nearly any data set, often in multiple ways. Gaussian density estimators applied to the pixels defining leaf outlines measures shape, allowing the genetic basis of the plant form to be studied. Geodesic distance captures the branching patterns of grapevine clusters, permitting the analysis of their evolution and modeling of berry development. We can analyze and compare the most persistent features in our barcodes, summarize them using the Euler characteristic, or truly calculate the overall topological similarity between barcodes using bottleneck distance. Using mapper, we can summarize the structure of data as a graph, and upon visualizing nodes of interest, identify the data points—whether voxels of an X‐ray CT scan or nodes corresponding to gene expression—for further study and interpretation.

**FIGURE 10:**
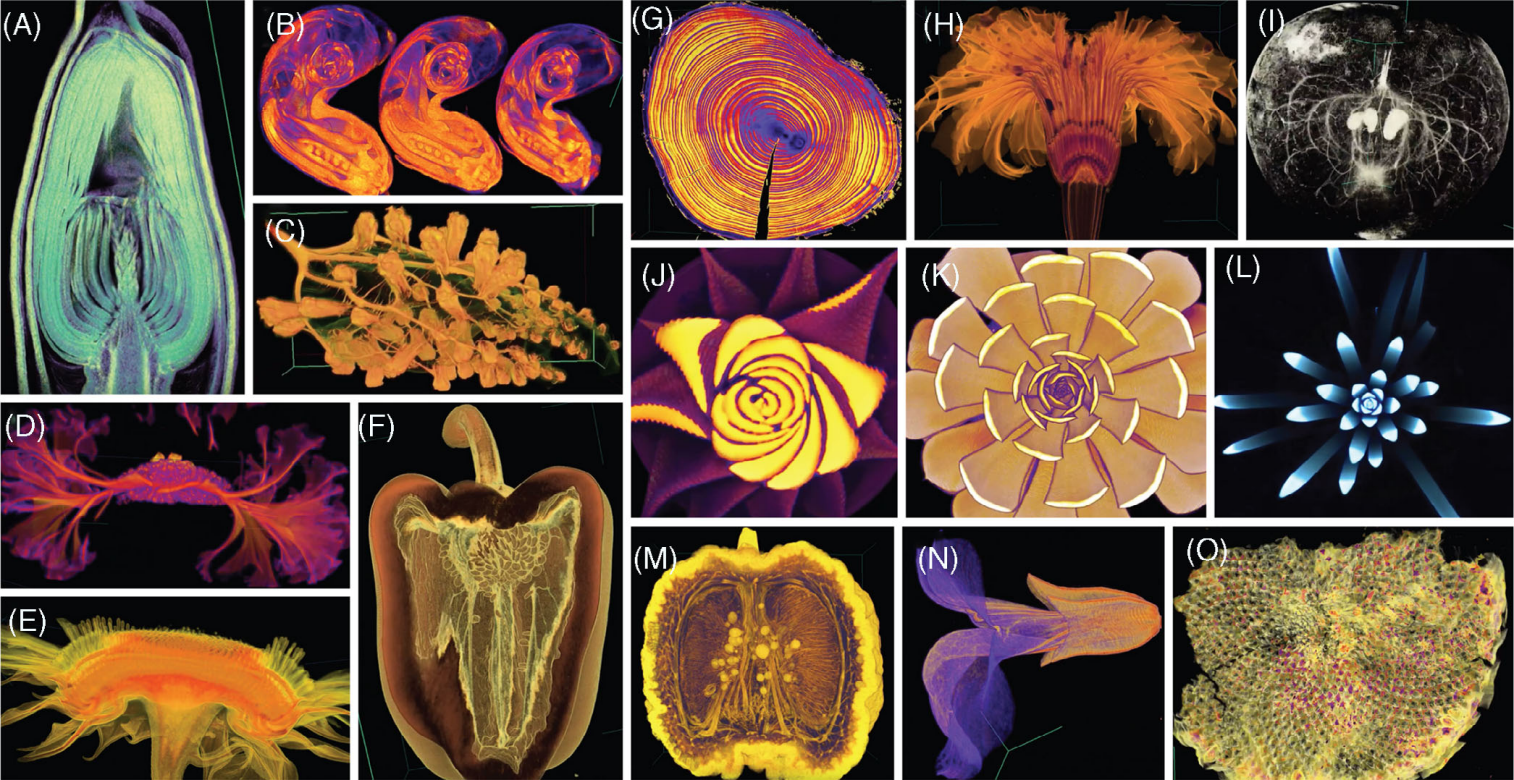
Endless forms most beautiful. X‐ray Computed Tomography (CT) scans of biological specimens showing the diversity of morphology in the natural world. A, Magnolia bud, B, bean flowers, C, grapevine leaf with phylloxera galls, D, the fasciated meristem of a velvet flower, E, side view of a sunflower disc, F, bell pepper, G, tree rings, H, marigold flower, I, vasculature within an apple, J, Haworthia, K, Echeveria, L, Agave hybrid, M, citrus fruit, N, monkeyflower, O, archaeological sunflower disc specimen

The promise of the application of TDA to biology is still in its infancy. Unlike any other method in biology, TDA provides a way to measure topological features and shapes in a comprehensive way. The versatility of filter function selection allows TDA to be applied to any number of data sets across sub‐disciplines: structural biology, evolution, molecular biology, medicine, neuroscience, and developmental biology. The methods described here can be applied to higher‐dimensional data sets that are dynamic or evolve over time,^58^, ^59^, ^60^, ^61^ easily accommodating biological complexity. Regardless of data size, complexity, or dimensionality, TDA provides concise summaries of the information content of any data set from the perspective of shape and structure. Given the spectacular diversity of form across biology (Figure 10), a method like TDA, that can be customized to measure shape using a tailored filter function, will allow previously unstudied phenomena to be analyzed from the perspective of shape. The vision of TDA, that data is shape and shape is data, will be relevant as biology transitions into a data‐driven era where the meaningful interpretation of large data sets is a limiting factor.

## AUTHOR CONTRIBUTIONS

**Erik Amézquita:** Conceptualization; writing‐original draft; writing‐review and editing. **Michelle Quigley:** Conceptualization; writing‐original draft; writing‐review and editing. **Tim Ophelders:** Conceptualization; writing‐original draft; writing‐review and editing. **Elizabeth Munch:** Conceptualization; writing‐original draft; writing‐review and editing.** Daniel Chitwood** **:** Conceptualization; writing‐original draft; writing‐review and editing.
